# The gendered dimensions of the anti-mask and anti-lockdown movement on social media

**DOI:** 10.1057/s41599-022-01442-8

**Published:** 2022-11-26

**Authors:** Ahmed Al-Rawi, Maliha Siddiqi, Clare Wenham, Julia Smith

**Affiliations:** 1 Simon Fraser University, 8888 University Dr., Burnaby, BC V5A 1S6 Canada; 2 London School of Economics and Political Science, Houghton St, London, WC2A 2AE UK

**Keywords:** Medical humanities, Health humanities

## Abstract

This paper examines the anti-mask and anti-lockdown online movement in connection to the COVID-19 pandemic. To combat the spread of the coronavirus, health officials around the world urged and/or mandated citizens to wear facemasks and adopt physical distancing measures. These health policies and guidelines have become highly politicized in some parts of the world, often discussed in association with freedom of choice and independence. We downloaded references to the anti-mask and anti-lockdown social media posts using 24 search terms. From a total of 4209 social media posts, the researchers manually filtered the explicit visual and textual content that is related to discussions of different genders. We used multimodal discourse analysis (MDM) which analyzes diverse modes of communicative texts and images and focuses on appeals to emotions and reasoning. Using the MDM approach, we analysed posts taken from Facebook and Instagram from active anti-mask and anti-lockdown users, and we identified three main discourses around the gendered discussion of the anti-mask movement including hypermasculine, sexist and pejorative portrayals of “Karen”, and appropriating freedom and feminism discourses. A better understanding of how social media users evoke gendered discourses to spread anti-mask and anti-lockdown messages can help researchers identify differing reactions toward pandemic measures.

## Introduction

Increasing evidence of the utility of face masks in reducing coronavirus transmission has normalized their use as an effective protective measure globally. However, scores of people choose not to wear a mask in public, stating different reasons for this decision. On August 29, 2020, for example, hundreds rallied in Paris to march against the newly introduced mask mandate (Pollet, 2020). An anti-mask event held in Vancouver, Canada included several hundred people gathered for a “freedom rally”, decrying ideas of mandatory mask-wearing (Little, 2020). Who defies public health recommendations regarding social distancing and mask-wearing is dependent upon a variety of overlapping factors, including age, gender, demographics, political beliefs and psychological traits (Cheng et al., 2020).

While social media provides powerful insights into the public’s response to the restrictions that accompany the pandemic, such as the mandate of face masks and enforced lockdowns, the number of studies on polarized public discourse based on gender remains limited. This paper attempts to conduct a thorough investigation into the gendered aspect of anti-mask and anti-lockdown discourse on social media. As the influence of social media remains significant, particularly during a prolonged pandemic like COVID-19, it is essential to investigate public discourse through a gendered lens.

### Gendered social media discourses

There has been little research done on gender differences in adherence to health agencies’ COVID-19-related directives. By gender, we refer to the socially constructed identities of men/boys, women/girls, and non-binary identities. Gender differences result from a variety of factors including socialization and biology, and gender roles and norms are often manifested through communication and culture (Rose et al., 2012).

Previous research suggests that women more often abide by health regulations as compared to men in many parts of the world (Masters et al., 2020). However, a French survey that aimed at studying the emotions behind the anti-mask movement showed some different results. The Jean-Jaures Foundation, a French think tank that conducted a study monitoring 1000 anti-maskers, found that people who reject mask-wearing are more likely to be older, with 50 being the average age, female (63%), and have a high degree of distrust in public institutions and social media (51%) (Bristielle, 2020). Also, a cross-sectional study conducted on US adults shows that there was no statistically significant difference in social-distancing behaviours exhibited by gender, urban city, race, monthly family income, or political affiliation (Masters et al., 2020). In addition, Kaspar (2020) studied motivations for social distancing through a health app and concluded that age and gender had no significant effect on the parameter. Further, Tobol et al. (2020) found insignificant gender differences between men and women when analyzing dishonest mask-wearing behaviours in Tel Aviv, Israel contrary to previous research that deem men as more accepting towards dishonesty than their female counterparts (Rawwas et al., 2004). Many studies have examined gender in relation to rule compliance. However, few studies consider the role of social media in communicating, reinforcing and propagating gender norms that defy public health regulations.

In this respect, we borrow Connell’s theory of hegemonic masculinity to ground our argument around the dominant behaviour of men in the context of the anti-mask movement. Connell (1987) describes how the normative idea of a “real man” is built upon male dominance and an inter-male hierarchy. In order to explore social media discourses around various genders, this paper draws from the normative ideas of masculinity and how it extends into the realm of gendered interactions. Toxic masculinity, according to Kupers (2005, p. 714), is a complex notion of socially regressive masculine qualities that encourage dominance and control, disrespect of women, discrimination towards sexual minorities, unwillingness to admit weakness and non-compliance to rules.

This study fills a gap in the literature as it examines the gendered aspects of the COVID-19-related anti-mask and anti-lockdown online community. In our survey of the literature, we found just a few studies that examined the issues of facemasks and lockdowns. For example, one study conducted a systematic sentiment analysis of Tweets collected from two prominent hashtags: #IndiaLockdown and #IndiafightsCorona. A total of 24,000 tweets were studied to gauge the feelings of Indians toward the lockdown. The results showed a positive reaction to the lockdown whilst the population entrusting the government’s decision amidst minor emotions of fear, sadness and disgust (Barkur and Vibha, 2020).

COVID-19 public discourses around the anti-mask and anti-lockdown issue on social media are important because they shed light on the nature of an online community that tries to disrupt public order and create confusion about vital public health guidelines. Social media remains one of the major outlets for this community to express itself and our study provides novel insight into the kind of gendered discussions on COVID-19 and the anti-mask movement. Indeed, social media is recognized as a significant resource for public health surveillance, especially in relation to infectious diseases (Leung and Nicoll, 2010).

A better understanding of the gendered aspects of the anti-mask and anti-lockdown communities can inform public health campaigns aiming to counter these messages. Using multimodal discourse analysis of Facebook and Instagram posts taken from active anti-mask and anti-lockdown users based on the number of times they post messages, we identified three main discourses around the gendered discussion of the anti-mask movement including hypermasculine, sexist and pejorative portrayals of “Karen”, and appropriating freedom and feminism discourses.

## Background: Masks, social distancing, and lockdowns

There is clear scientific evidence that supports the use of facemasks and enacting social distancing. Whether for a medical or non-medical grade, facemasks are endorsed by all health authorities as a way to limit infection as they form a layer of defence by filtering viral particles such as aerosols and droplets (Van der Sande et al., 2008). The use of facemasks and other non-pharmaceutical features remains warranted with emerging pandemics like COVID-19 (Cheng et al., 2020). As masks are a non-pharmaceutical intervention measure to combat the virus without any discernible change in social practices, studies show that effective combinations of social distancing and mask-wearing can significantly flatten the epidemic curve (Li et al., 2020). Many researchers believe that the importance of mask usage is under-emphasized and needs sufficient publicity and encouragement from trusted sources (Wang et al., 2020). Community-wide benefits are maximized when masks are used with social distancing, and there is high nationwide compliance (Eikenberry et al., 2020).

In many countries, wearing masks has a cultural effect as well. For instance, it is a symbol of solidarity in China (Lynteris, 2020). In many other Asian countries such as South Korea and Japan, masks were common even before the COVID-19 outbreak, owing to previous histories of outbreaks including SARS, MERS and seasonal influenza (Haelle, 2020). On the other hand, most European and North American countries associate masks with “unwellness” (Feng et al., 2020). However, additional contextual factors have to be considered in this regard, such as affordability and availability. In addition to this, the public needs to practice the proper usage of masks along with other hygiene measures, such as handwashing, and there are many cultural factors involved (Tso and Cowling, 2020). Arguably, the improper use of facemasks could nullify the protective effect and even increase the risk of infection, for example in the case of not changing disposable masks (Feng et al., 2020).

Those opposed to masks often termed ‘anti-maskers’, present a broad spectrum of reasons to oppose public authorities’ recommendations concerning masks; some view them as annoying or associate them with conspiracy theories (Stewart, 2020). Many anti-maskers also believe that masks are detrimental as they make the person wearing them inhale their own carbon dioxide. For anti-maskers, the perceived state’s mandate aims to enslave the human population and their job is to challenge this perceived “dictatorship” (Allemandou, 2020). In brief, the aversion to mask-wearing speaks to an underlying mistrust in public health messaging and the science around COVID-19 (Hapuhennedige, 2020).

It is sometimes considered unmanly to cover the face (E-MASK-ulating) as it shows weakness (Vershbow, 2020). A few studies indicate that women show more tendency to wear masks than men hence they tend to have a higher risk perception towards COVID-19 (Okten et al., 2020). Also, a higher percentage of women who believed masks have a role in society voluntarily wore a mask when shopping, compared to the percentage of men (Bir and Widmar, 2021). A study conducted in Taiwan in February 2020 showed that women are 16 percent more likely to wear masks, although protective behaviour in both men and women drops significantly when they are socializing in groups (Chuang and Liu, 2020). Similarly, Haischer et al. (2020) conducted a study on shoppers entering retail stores during periods of June- August 2020 and found only 41% wearing masks out of which females had a 1.5 times greater tendency to do so. Women also followed greater rules of social distancing as compared to men (Okten et al., 2020; Pedersen and Favero, 2020). Women exhibit higher adherence to diligent hand-washing practices and personal protection than men who dismiss the pursuit as useless or time-consuming (Guzek et al., 2020). Men have been found to follow rules of social distancing less consistently because of their proclivity to be more involved in sports and physical activity (Sekulic et al., 2020). These findings are in line with previous studies that place women within the ambit of rule-abiding individuals (Coffé and Bolzendahl, 2010).

As with masks, physical distancing measures significantly decrease the risk of person-to-person transmission by reducing droplet transmission that occurs within 3–6 ft, especially in closed workplaces (Bischoff et al., 2013). Many countries have struggled to balance physical distancing measures with economic and social costs, with sharply divergent views between those arguing for a resumption of economic activity and those arguing for lockdowns to enforce physical distancing (Caulkins et al., 2020). While lockdowns have succeeded in “flattening the curve”, they come at a high price; the global economy has experienced the highest-ever recorded recession (Caulkins et al., 2020). Decision-makers face a conundrum- ease physical distancing measures in order to mitigate economic hardships and risk overwhelming the healthcare care system, or prioritize public health at the expense of the economy—although this dichotomy in itself oversimplifies that challenge.

This is particularly true as lockdowns can contribute to non-pandemic-related public health needs. Many studies demonstrated elevated levels of anxiety and major depression induced by the lockdowns (Patsali et al., 2020), particularly among women (ECDC, 2020). Indeed the effects of lockdowns are particularly gendered with associated increased rates of interpersonal violence (Mittal and Singh, 2020), reduction in access to sexual and reproductive health services, increased care work forcing women to give up paid employment, and higher job losses in female-dominated sectors like service and tourism (Wenham et al., 2020; Azcona et al., 2020).

Based on the above framework and discussion, our study attempts to answer the following research question: What are the gendered discourses around anti-mask and anti-lockdown measures on Facebook and Instagram?

## Methods

In this study, we used multimodal discourse analysis (MDM) which is an extension of discourse analysis that uses the study of language in combination with images, scientific symbolism, gesture, music and sounds (O’Halloran, 2011). It is a method of diverse analysis that looks at not just how individual modes communicate, but also at how they interact with one another to create semiotic meaning. The method employs both visual and textual analysis and focuses on appeals to emotions and reasoning (Hahner, 2013; Joffé, 2008). Researchers then try to isolate patterns in these elements along with hashtags associated with the posts, classifying them into main discourses or themes. This method is highly effective to analyse small samples from social media like videos, posts and memes as it presents a deeper analysis into both the textual as well as the visual representation and what the contexts entail. Researchers need to examine different dimensions including the content, form, and function, which are often intertwined (Kress, 2012).

The analysis is primarily focused on the North American discourse but has considerable relevance to global issues as the dataset includes posts from all over the world due to the nature of social media that allows anyone to post from anywhere. Our focus was unabridged on the diverse content that we examined in the dataset.

As health communication is an active topic on social media platforms, such as Facebook, many researchers use this method to conduct an in-depth and exhaustive analysis of related samples. Hunt (2015), for example, studied multimodal discourses in 72 Facebook posts to analyse pages disseminating information regarding diabetes from two popular diabetes-related Facebook pages. The analysis infers from visual and etymological features of social relationships demonstrated through these posts, and three overarching strategies that were consistent in the discourses around these pages. Another relevant study analysed information regarding vaccines against communicable diseases like measles (Ma and Stahl, 2017). Using Facebook data, the researchers collected 122 posts with a total of 1456 total comments from January 2015 to August 2016, after the 2014 California measles outbreak and consequently the 2015 California measles outbreak. These posts were then divided into textual (text-only) and graphic (images and videos) material for multimodal critical discourse analysis which then presented relevant characteristics towards the propagation of the posts (Ma and Stahl, 2017). The researchers used a systematic analysis method based upon a three-layer model of critical discourse analysis: “description (textual and graphic analysis), interpretation (process of discursive practice), and explanation (analysis of social practices)” (Ma and Stahl, 2017, p. 306).

Further, in a study to investigate the language used in fitness industry advertising, Zjakic et al. used multimodal analysis on a sample of 120 advertisement posts from the Facebook pages of three popular gyms in Australia. Out of the 120, at least 85 contained an imperative verb, while “93% of advertisements incorporated visual and verbal elements to convey its meaning (i.e. 79 of 85)” (Zjakic et al., 2017, p. 17). Also, Nilsen (2020) used online observation and multimodal analyses of modes and semiotic resources found in Facebook profiles to study the types of discourses employed by European women supporters of ISIS analyzing the ideologies used to set up compliant Facebook profiles. Their analysis uses different modes like photos, posters and texts with semiotic resources like vocabulary, symbols, clothing and colours in the posts. Similarly, Hakoköngäs et al. (2020) studied a sample of 426 memes posted on Facebook by two far-right groups in Finland. The singular meme could have characteristics from several other themes such as history, humour, mythology, symbols, news and mottos (Hakoköngäs et al., 2020).

**Fig. 1:**
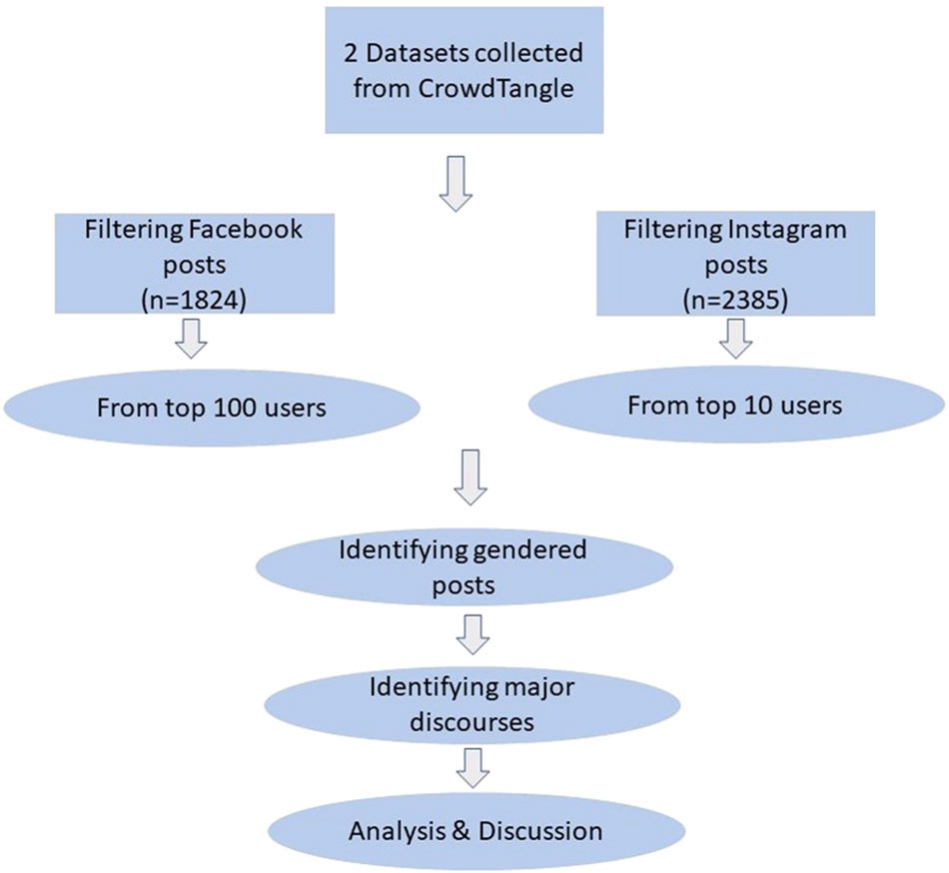
Flowchart showing the selection of the social media users and content for the study.

During the first round of analysis, we generated initial notes describing the multimodal contents of the posts after which they were classified into broader discourses based on their content and context. The primary meaning was defined by considering all the components of the posts such as their visual and written text component. It is pertinent to mention here that the data we collected and analysed was in English based on the search terms we used. Due to our ignorance of other languages, we did not include non-English posts in the analysis. To identify the relevant Facebook and Instagram posts, we downloaded references to the anti-mask and anti-lockdown posts using 24 search terms in CrowdTangle (Figshare, 2021). The items we identified were posted between January and October 2020. These search terms are: ‘nolockdown’, ‘nomask’, ‘nosocialdistancing’, ‘nofacemask’, ‘nosocialdistance’, ‘maskoff’, ‘facemaskoff’, ‘NoNewNormal’, ‘removethemask’, ‘NoMaskOnMe’, ‘Antimasques’, ‘antimask’, ‘NoMasksForKids’, ‘endlockdown’, ‘Saynotomasks’, ‘MasksDontWork’, ‘nofacemask’, ‘RemoveTheMasks’, ‘MasksOff’, ‘nomasksonourchildren’, ‘NoMoreMasks’, ‘NeverMasker’, ‘bareface’, ‘BareFaceIsLegal’. Two researchers examined a sample of 2385 Instagram posts (about 25% of the total sample) and 1824 Facebook posts (over 48% of the total sample) taken from the most active anti-mask and anti-lockdown users in terms of the number of their public posts on the two social media platforms. The data retrieved from the two social media platforms was reorganized based on the descending order of the frequencies of users’ posts. The users with the highest number of social media posts were put at priority with the highest first and so forth until the analysis was completed. Only the anti-masker posts were included in the analysis as many other users, especially on Facebook, were not involved in the anti-mask movement but were only referencing or criticizing it (Fig. 1).

We chose both Facebook and Instagram because they are two of the world’s largest social media platforms (Smith and Sanderson, 2015) and are seen to be having sustainable growth in the tech industry (Lai and Yang, 2014). Besides choosing these specific platforms due to their popularity for this study, the key affordances of both are comparable. Both platforms allow image and video sharing. Moreover, users can also share posts and stories, leave a comment, or like someone’s post on both platforms. Alongside that, the possibility of adding a location on both platforms is also available. Since both services offer interactions through common elements of text, sound and visual elements, the data we collected makes for a very thorough yet detailed sample to investigate. Therefore, common gratifications (social interaction, entertainment, passing time, browsing, need for recognition), as well as technology attributes (visual element, recommendation algorithm, privacy setting, openness, simultaneousness) of Facebook and Instagram (Kim and Kim, 2019), make them viable platforms for our study since they offer users different means to share information and news. For research purposes, this allows researchers to have richer content than exclusively examining textual content.

From a total of 4209 social media posts, the researchers manually filtered the explicit visual and textual content. In this process, the researchers managed to identify 297 posts in total that visually showed men, women, or non-binary people and/or directly referenced textual content pertaining to different genders: 215 Instagram posts from 4 users and 82 Facebook posts from 20 users (Figshare, 2021). For Instagram, the researchers examined the top 10 users who are far more active than Facebook users in posting anti-mask messages. On the other hand, the researchers needed to examine the top 100 Facebook users to identify relevant posts. We emphasize here the need to pay more attention to Instagram as it seems to be a vital platform for anti-maskers and other users who engage in fringe issues (Al-Rawi, 2019, 2021). The other possible reason could be due to the apparent lack of strict content moderation on Instagram unlike the case of Facebook.

It is important to mention here that there are numerous other posts that are not included in this study because they did not directly feature or involve any gendered references. After several rounds of deliberation and discussion by two researchers, three main discourses emerged including hypermasculine, sexist and pejorative portrayals of “Karen”, and appropriating freedom and feminism discourses. The pejorative memetic image of the white privileged female ‘Karen’ has risen to popularity in recent months, shifting from social media jargon to mainstream usage at a time of intense public health and racial social justice discussions (Negra and Leyda, 2021). The ‘Karen’ stereotype combines a specific definition of entitled white supremacy and class privilege into toxic sexism and misogyny. This discourse is extremely essential to our findings as it organizes a specific type of women’s identity (MacKinnon, 1991) into a distasteful and pejorative category. The gendered overtones that the discourse entails support Connell’s (1987) and Kupers’ (2005) conceptualization of the degradation of the female figure in order to mask potential misogyny.

The theoretical framework that we use in this study is based on a feminist paradigm that limits the scope of analysis, and the discourses that have emerged from our analysis present a proclivity towards hypermasculine criticism. However, the major discourses have been inductively identified after multiple rounds of review and discussions amongst two researchers in order to minimize any prejudgement. These discourses emerged from the data analysis though it is difficult to avoid potential bias in the discourse identification process. In addition, minor discourses or themes might have been overlooked.

## Results

To answer the study’s research question, the multimodal discourse analysis around the gendered social media posts by anti-maskers showed three main discourses that are explained in detail below.

### Hypermasculine discourse

Hypermasculinity can be defined as the exaggeration of male stereotypical behaviours more or less adhering to a sociological gender construct (Hickey, 2016). This term often reflects extreme stereotypical gender roles like physical strength and overt sexuality, while men on the opposite spectrum are rendered weak and disempowered (Matson et al., 2019). In this respect, Connell’s gender order theory, which recognizes multiple masculinities that vary across time, culture and the individual, is relevant here. Conceptually, hegemonic masculinity can explain how and why men maintain dominant social roles over women and other gender identities which may include non-binary gender groups, who are often perceived as “feminine”. The macho aura of the “man” is evident in many social media posts that we studied.

As Connell points out, masculinities are not exactly equivalent to men, but they concern the *position* of men in a gender order. It becomes, therefore, inevitable for us to view these points through the analysis of patterns and social practices with which people (both men and women, though predominantly men) engage (Connell, 2005). As the theory abundantly places emphasis on women having a role in shaping masculinities, it is interesting to note differences between this and the other important gendered discourses.

**Fig. 2:**
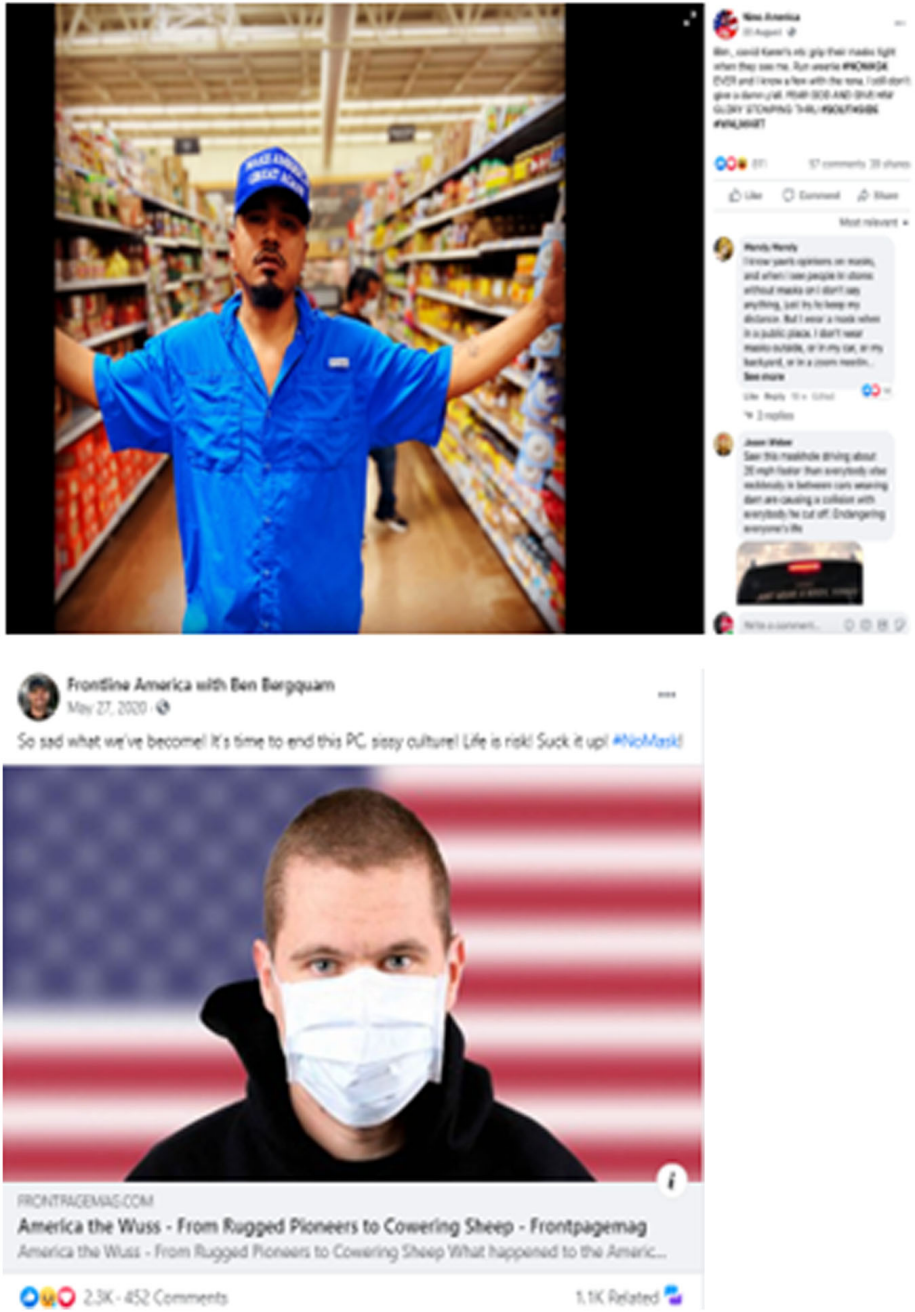
Social media posts representing hypermasculine discourses.

This social media discourse is the most dominant in both Instagram and Facebook, and it constitutes posts referencing hypermasculine men who appear to resist complying with rules designed to curb the spread of the virus-like using masks and following social distancing guidelines. The gendered dimension of these posts clearly shows that men are invincible since COVID-19, if it is not a hoax, cannot affect them because they are strong and emotionally, physically, and intellectually independent. Users who post such messages often view the others who abide by public health authority guidelines as passive, meek or as Fig. 2 shows a ‘wuss’. To man up, they claim that men have to resist health agencies’ guidelines that allegedly violate their freedom, ruin the economy, and create more harm than good. Many of these posts show support for conservative figures especially former US President Donald Trump and rejection of what is perceived as liberal propaganda about the pandemic. These sentiments are often expressed in the use of hashtags like #ChinaVirus, #MAGA, and #Trump2020. This finding closely aligns with previous studies that referenced the connection between anti-mask or anti-pandemic community and support for Trump and/or conservative views (Gonzalez et al., 2021; Grunawalt, 2021; Mahalik et al., 2022; Taylor and Asmundson, 2021).

### Sexist and pejorative portrayals of “Karen” discourse

This kind of pejorative discourse includes posts that reference women perceived as privileged, often negatively labelled as “Karens” who are almost always represented as overemotional. The term “Karen” is a pejorative expression currently used in popular media to typically describe a “middle class” white woman whose dismissive and entitled behaviour stems from white privilege (Nagesh, 2020). As the word “Karen” is still vague due to its recent use, it is essential to define it to establish clarity around why the word and concept generated importance for our paper. “Karen” has, in recent years, become a widespread term referencing a specific type of middle-class white woman, who exhibits behaviours that stem from privilege. “Karen” is associated with the kind of person who demands to “speak to the manager” in order to belittle service industry workers, is anti-vaccination, and [often] carries out racist micro-aggressions, such as asking to touch black people’s hair” (Nagesh, 2020). With the name occupying stronger spaces in mainstream media, especially social media platforms, an entire debate is spun around the controversial and problematic nature of this word. Interestingly, the coronavirus pandemic has spun a dichotomous narrative around “Karen”, as it is used both for a woman who blatantly flouts rules and refuses to wear a mask as well as someone who regularly reports anti-maskers to law enforcement authorities (Romano, 2020; Lewis, 2020). Most recently, the discourse has been increasingly used on social media like Reddit where offensive and pejorative comments are an issue of concern. During the pandemic, the word started off as “Toilet paper hoarding” women and then shifted to a new species of “Karen”—a white woman who detests the lockdown and social distancing rules because she needs a haircut (Tiffany, 2020). The anti-maskers, however, use the word “Karen” to refer to any woman who criticizes their actions. Reflecting on Connell’s (1987) theory of hegemonic masculinity, this discourse strongly ties back to problematic male behaviour towards females. The “Karen” term highly legitimizes offensive and casual sexism against women who choose to challenge authority. Women are discriminated on the same behaviour that entitles men to be exempted from it.

**Fig. 3:**
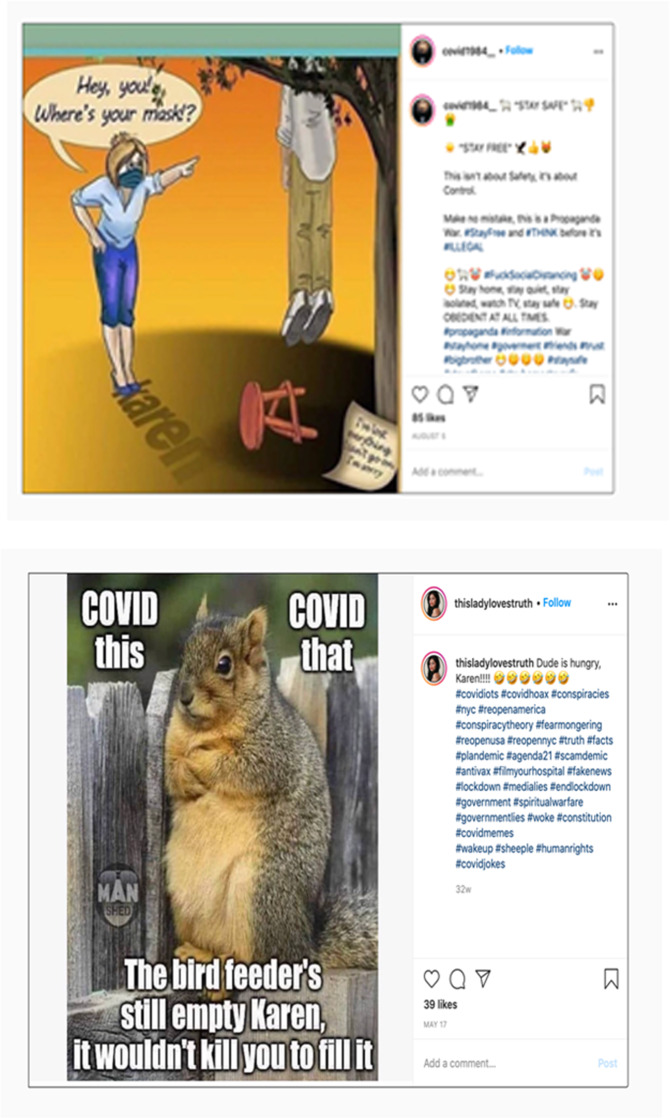
Social media posts representing pejorative portrayals of “Karen” discourses.

In this discourse, there is a clear political attack against public health authorities often associating them with leftists or liberals who are unjustifiably complaining about anti-masking/anti-vaccine users. Some of the posts mock public authorities and their figures as a way to discredit their claims. Figure 3, for example, shows a squirrel talking to a Karen including several hashtags which clearly consider the pandemic as a hoax such as #agenda2, #plandemic which is a reference to the documentary on COVID-19, #covidhoax, #endlockdown, #medialies, #scamdemic, and #fakenews. It is important to note here that both conservative and liberal social media users attack the figure of “Karen” for opposite ideological reasons (Bhasin et al., 2020), as stated above. For the left, a “Karen” is someone who refuses to wear a mask and social distance, unlike for the right a “Karen” complains about those who do not wear masks or social distance.

These users mostly identify as conservative nationalists whose mission is to oppose what they perceive to be oppressive authority rules that infringe on their freedom of expression and/or movement. What is implied here is a general intellectual superiority, as the progressive liberals are viewed as less knowledgeable or aware of the alleged schemes to control people’s lives. In other words, the polarized political dimension plays a major role in enhancing this kind of discourse. There is also a clear focus on women (Karens) who are presented as aggressively complaining about anti-maskers. Men are not “Karens” even though they make similar complaints, denoting the sexist dimension in this pejorative term. Though it is difficult to know the identity of online users only from their public profiles, we found social media evidence based on users’ profiles and posts, suggesting that such pejorative labels often come from both men and women users mostly due to ideological reasons.

### Appropriating freedom and feminism discourse

**Fig. 4:**
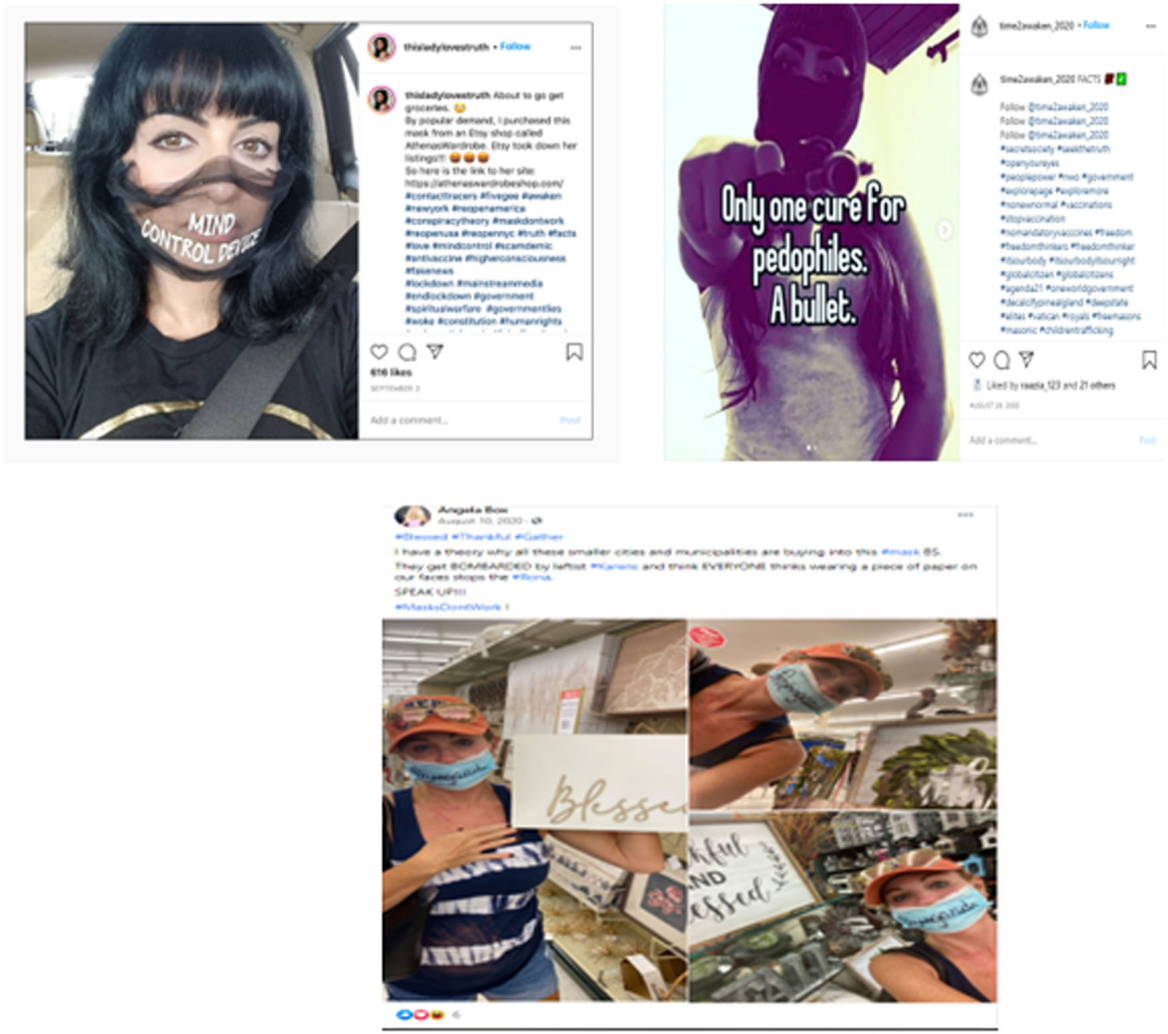
Social media posts representing the third discourse on appropriating freedom and feminism.

This discourse includes posts mentioning women who project toughness, defiance, humour along with a tendency to reject rules including the use of unusual anti-lockdown measures. There is also a clear connection between anti-mask or anti-lockdown views and conspiracy theories by appropriating feminism and freedom of expression issues. Figure 4 shows an association made between measures taken by health agencies and conspiracies about alleged schemes to control people’s lives or about alleged liberal paedophiles on the loose such as using terms like Pizzagate, childrentrafficking, and Pedogate. One post shows the word “propaganda” written on the woman’s facemask to show her mistrust in health agencies’ guidelines, while the accompanying text mentions “Rona”, slang for COVID-19, with the intent to discredit the pandemic and related science. Again, the examination of the accompanying hashtags is useful because they provide insight into the meaning of these posts. For example, we can find terms like #CoronaFascism and #ChooseLife to stress the importance of freedom and independence from public authority regulations that are perceived to be oppressive and meaningless. This is also echoed in the use of other hashtags such as #RiseUp, #FightBack, #Tyranny, #ReopenAmerica, and #ReopenSchools.

**Fig. 5:**
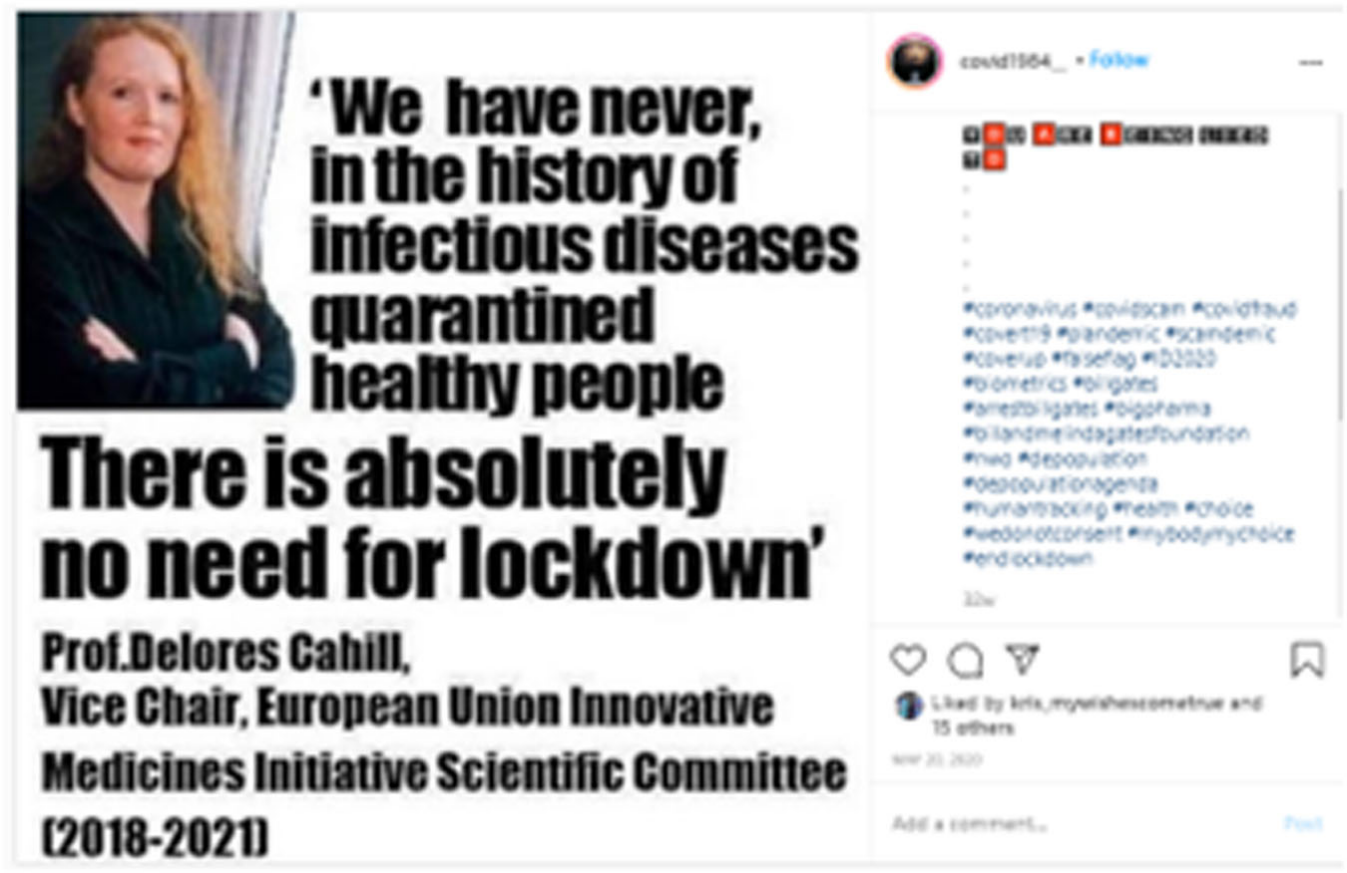
Instagram post discussing freedom of choice.

Interestingly, many women users in this community are appropriating popular terms from feminist movements around abortion rights, sexual freedom, and reproductive rights like #wedonotconsent, #mybodymychoice, #itsourbody, #itsourbodyitsourright, and #choice (see Fig. 5). One Facebook user, for example, mentions the following about the government’s use of contact tracing apps: “[It] takes us to an entirely new level of invasiveness, in which we no longer have any control of who has information about us and what that information is being used for,” ….The question is, who owns your body?” This aligns with the previous discussion on the concerns this community often expresses about agency as well as freedom of choice and expression, yet the terms and arguments they are using are borrowed from previous social movements around women’s rights and social justice.

## Discussion

Our results both confirm and advance previous findings regarding gender norms and public health compliance. Some studies referenced how men, more than women, agree that wearing a face covering is shameful, not cool, a sign of weakness, and a stigma; and these gender differences also mediate intentions to wear a face covering (Zanin et al., 2021). Scholars have argued that the resistance to mask-wearing may be rooted in masculinity and the desire to appear strong or “tough”. For example, a study conducted in June 2020 associated masculine toughness with higher negative feelings toward mask-wearing (Palmer and Peterson, 2020). As we have further demonstrated, hypermasculinity leads to a sizeable hindrance to the normalization of mask-wearing (Bhasin et al., 2020) as individuals with hyper-masculine identities often assert their self-perceived strength through a lack of fear of getting sick and non-compliance-to-guidelines related to avoiding hand washing and social distancing (Cassino and Benson-Cassino, 2020). This discussion is directly related to Connell’s (1987) theory of hegemonic masculinity, a conceptual framework that can effectively explain some men’s anti-pandemic conduct.

The analysis of pejorative portrayals of “Karens” demonstrates how values and beliefs around gender influence social norms regarding masking. Similar to Bhasin et al. (2020) analysis of pejorative “Karen” memes on Twitter—which finds “Karen” is more often referenced by anti-maskers compared to pro-maskers but is always heavily gendered—our analysis demonstrates anti-mask movement members show clear animosity against “Karens” and/or health authorities, often associating ‘Karens’ with various conspiracy theories and as being useless, senseless and intellectually inferior. On the other hand, women who belong to the movement are shown in the memes appropriating feminist discourses as strong, defiant, funny, and in full control of their lives. The juxtaposition of these two types of women reflects both negative and positive gendered stereotypes.

This paper has many limitations. First, our study focused on two social media platforms, and it does not claim to fully explore the gendered dimensions of the anti-mask and/or anti-lockdown movement because there are different other sites where this online community interacts such as YouTube, Twitter, Reddit, and Telegram. Second, we examined a limited number of visual and/or textual social media posts that explicitly feature and/or mention different genders as there could be other indirect or implicit references to gender that we could have missed. In addition, we only analysed English language posts that were overwhelmingly dealing with the US context; hence, there is a need to further understand the gendered dimension of this online community in other countries and languages. A very important point to make for possible future studies is to focus on more diverse possibilities that envision global content rather than the one which is heavily centred around North American discourses by using different search terms that take into account language differences. This may allow for a manifold audience and a wholesome dialogue around the same topic. Finally, our methodological approach is limited to identifying major discourses and could have some potential bias in terms of overlooking minor issues, so using other communication research methods like content analysis can be useful.

## Conclusion

Studying the gendered discussion around COVID-19 and anti-pandemic measures can assist in better understanding the nature of the communities that engage in such discourses on social media sites like Facebook and Instagram. For example, the relationship found here between hypermasculinity and anti-mask/anti-lockdown discourse is particularly concerning, and suggests the need for more research in this area to further examine those users propagating such ideas and others susceptible to such messaging. There is also evidence that many anti-mask users belong to conservative circles, for they seem to be motivated by an anti-liberal political agenda. The continued sexist portrayal of “Karen” across the spectrum suggests this trope is unhealthy and unhelpful. Understanding the ways social media users evoke gendered discourses to spread anti-mask and anti-lockdown messages can also help researchers gain important insight into the differences between men and women who often attack others that abide by pandemic measures.

## Data Availability

The dataset analysed in this study has been uploaded into the Figshare repository (Figshare, 2021).
